# Supplementary material for: Content-rich biological network constructed by mining PubMed abstracts
Source: BMC Bioinformatics. 2004 Oct 8;5:147. doi: 10.1186/1471-2105-5-147 (PMC528731; doi:10.1186/1471-2105-5-147)
Supplement: Additional File 2 — The original results of the above study (non-essential files are deleted to keep the file size under the limit set by BMC bioinformatics). [file 1471-2105-5-147-S2.bz2 › chilibotAdditionalFile2/dip50/235ID8006584E759/html/IL2RB_IL2.html]

 


 **IL2RB** and **IL2** 
  
Found 3819 abstracts in PubMed, retrieved 50.  
 

 What does Google say? 
 PDF only 
| .edu only 

---

**Interactive relationship** (e.g. stimulation, inhibition, etc)

**Neutral relationship**- BACKGROUND Daclizumab 150 kD, a humanized monoclonal antibody mAb against the alpha chain of the membrane bound interleukin 2  [ **IL2** ]  IL 2  [ **IL2** ]  receptor  [ **IL2RB** ]  IL 2R also binds soluble interleukin 2R alpha sIL 2Ralpha.  Ref: 12846768 Kidney Int, 2003

**Non-interactive relationship** (e.g. studied together, co-existance, homology, etc.)

- IL 2  [ **IL2** ]  receptor  [ **IL2RB** ]  expression was measured by immunofluorescence and flow cytometry.  Ref: 12780688 Clin Exp Immunol, 2003
- Although IL 2  [ **IL2** ]  receptor  [ **IL2RB** ]  signaling is involved in activation of NK cells, neither the mechanism for this activation nor the role of EtOH consumption in modulating activation is completely understood.  Ref: 12700414 Toxicol Sci, 2003
- SHB mediates certain responses in platelet derived growth factor PDGF receptor, fibroblast growth factor FGF receptor, neural growth factor NGF receptor TRKA, T cell receptor, interleukin 2  [ **IL2** ]  IL 2  [ **IL2** ]  receptor  [ **IL2RB** ]  and focal adhesion kinase FAK signaling.  Ref: 12776987 Curr Mol Med, 2003
- Main pro inflammatory cytokines culminated within 24 hours from the onset of surgery, marked elevations were noted in IL 1ra and the soluble IL 2  [ **IL2** ]  receptor  [ **IL2RB** ] .  Ref: 12688152 Sb Lek, 2002
- However, anergic T cells express the IL 2  [ **IL2** ]  receptor  [ **IL2RB** ] , and anergy can be broken by exogenous IL 2  [ **IL2** ] .  Ref: 12670403 Immunol Rev, 2003
- IL 2  [ **IL2** ]  receptor  [ **IL2RB** ]  expression was reduced after CD3 ligation, yet normal in numbers and affinity after PHA stimulation.  Ref: 12780688 Clin Exp Immunol, 2003
- and those undergoing rapid withdrawal of steroids using Simulect R interleukin 2  [ **IL2** ]  IL 2  [ **IL2** ]  receptor  [ **IL2RB** ]  inhibitor N = 9.  Ref: 12753323 Kidney Int< MedlineTA>Kidney Int, 2003
- We investigated whether blockade of the high affinity interleukin IL 2  [ **IL2** ]  receptor  [ **IL2RB** ]  effectively prevented T cell alloreactivity in cardiac transplantation.  Ref: 12660501 Transplantation, 2003
- The pathological changes of the graft, IL 2  [ **IL2** ]  receptor  [ **IL2RB** ]  expression in lamina propria lymphocytes, serum IL 2  [ **IL2** ]  concentrations, results of spleen lymphocytes transformation test and the IL 2  [ **IL2** ]  secretion capacity were determined and compared.  Ref: 12875685 Chin Med J (Engl), 2003
- Here, we report that detection of sIL 2R using the Immulite Immunoassay system in serum samples of patients who are treated with basiliximab, a monoclonal antibody against the alpha chain of the IL 2  [ **IL2** ]  receptor  [ **IL2RB** ] , may lead to false measurements.  Ref: 12667677 J Immunol Methods, 2003
- Abnormal IL 2  [ **IL2** ]  receptor  [ **IL2RB** ]  levels in non pregnant women with a history of recurrent miscarriage.  Ref: 12832383 Hum Reprod, 2003
- The concentration of soluble IL 2  [ **IL2** ]  receptor  [ **IL2RB** ]  R protein was measured in BAL fluid using ELISA as well, as an indicator of lymphocyte activity.  Ref: 12869037 Clin Exp Immunol, 2003
- We detected an upregulation or de novo synthesis of interleukin 1alpha IL 1alpha, IL 1beta, IL 8, macrophage inflammatory protein 1alpha MIP 1alpha, MIP 1beta, GRO alpha, transforming growth factor beta, MCP 1, IL 2  [ **IL2** ]  receptor  [ **IL2RB** ]  gamma IL 2Rgamma, IL 10Ralpha, and IL 6R.  Ref: 12874345 Infect Immun, 2003
- Measurements included total cholesterol, low density lipoprotein cholesterol, high density lipoprotein cholesterol, triglyceride, interleukin IL 6, soluble IL 2  [ **IL2** ]  receptor  [ **IL2RB** ] , and soluble necrosis factor p55 and p75 receptors.  Ref: 12792232 J Burn Care Rehabil< MedlineTA>J Burn Care Rehabil, 2003
- At 7 days postinfection, lymphocytes from italic Listeria italic infected rats showed significant increases in CD4 superscript superscript and CD8 superscript superscript cell counts and the CD8 superscript superscript CD4 superscript superscript ratio and exhibited increased production of IFN gamma and IL 2  [ **IL2** ]  receptor  [ **IL2RB** ]  expression compared with the noninfected control.  Ref: 12676610 Environ Health Perspect, 2003
- IL 2  [ **IL2** ]  receptor  [ **IL2RB** ]  alpha deficient IL2Ralpha mice spontaneously accumulate vast numbers of intestinal lamina propria LP T cells and develop bowel inflammation.  Ref: 12702495 Am J Physiol Gastrointest Liver Physiol< MedlineTA>Am J Physiol Gastrointest Liver Physiol, 2003
- CONCLUSION The monoclonal antibody against IL 2  [ **IL2** ]  receptor  [ **IL2RB** ]  alpha chain CD 25 has a marked effect upon severe steroid refractory GVHD.  Ref: 12812665 Zhonghua Yi Xue Za Zhi, 2003
- The inhibitory effect on human T cells was assessed and demonstrated at the level of proliferation DNA synthesis, IL 2  [ **IL2** ]  secretion, and IL 2  [ **IL2** ]  receptor  [ **IL2RB** ]  CD25 upregulation.  Ref: 12835898 Inflamm Res, 2003
- The serum cytokine levels of each patient group B LAHS versus T NK LAHS were expressed as the ratio of the median to the upper normal values of the respective cytokines and were as follows 19.05 versus 13.99 for soluble interleukin 2  [ **IL2** ]  IL 2  [ **IL2** ]  receptor  [ **IL2RB** ] , 0.67 versus 0.67 for granulocyte macrophage colony stimulating factor GM CSF, 0.64 versus 1.26 for G CSF, 5.70 versus 3.61 for M CSF, 1.54 versus 3.39 for interferon gamma IFN gamma, 13.17 versus 1.17 for IL.88 versus 1.58 for tumor necrosis factor alpha TNF alpha, 0.71 versus 0.41 for IL 1beta, 1.99 versus 0.21 for IL 12, and 105.32 versus 29.65 for IL 10.  Ref: 12731674 Int J Hematol, 2003
- CONCLUSIONS IL 2  [ **IL2** ]  receptor  [ **IL2RB** ]  blockade is effective for preventing alloreactivity and high grade rejection in cardiac transplantation.  Ref: 12660501 Transplantation, 2003
- Mechanistic studies revealed that stimulation of the proliferative response of lymphocytes by oxihumate is associated with an increased production of IL 2  [ **IL2** ] , as well as expression of the IL 2  [ **IL2** ]  receptor  [ **IL2RB** ]  in the setting of decreased production of IL 10.  Ref: 12710739 Z Naturforsch C < MedlineTA>Z Naturforsch C , 2003
- When we compared mean nocturnal levels, CD16 and CD25 T and B activated lymphocytes with expression of the a chain of IL 2  [ **IL2** ]  receptor  [ **IL2RB** ]  levels were higher, while CD8, CD8bright, CD20 total B cells, TcRd1 epitope of the constant domain of d chain of T cell receptor 1 and dTcS1 epitope of the variable domain of d chain of T cell receptor1 levels were lower in cancer patients.  Ref: 12797908 Int J Immunopathol Pharmacol< MedlineTA>Int J Immunopathol Pharmacol, 2003
- METHODS Thrombin antithrombin III complex TAT, plasma D dimer, serum type III procollagen peptide PIIIP, serum soluble IL 2  [ **IL2** ]  receptor  [ **IL2RB** ]  sIL 2R, Free tissue factor pathway inhibitor TFPI, and soluble intercellular adhesion molecule ICAM 1 were measured preoperatively around the same period when computed tomography CT was taken in 17 patients with AA mean age 72.2 years.  Ref: 12829084 Eur J Cardiothorac Surg, 2003
- Presentation by these migrants sustained expression of the IL 2  [ **IL2** ]  receptor  [ **IL2RB** ]  and promoted delayed type hypersensitivity.  Ref: 12871638 Immunity, 2003
- In this study, the authors observed that combined use of IL 18 and low dose IL 2  [ **IL2** ]  synergistically promoted in vitro proliferation of natural killer cells with up regulation of IL 2  [ **IL2** ]  receptor  [ **IL2RB** ]  alpha and also synergistically stimulated cytolytic activity and interferon gamma production by these cells.  Ref: 12806277 J Immunother< MedlineTA>J Immunother, 2003
- The serum level of soluble IL 2  [ **IL2** ]  receptor  [ **IL2RB** ]  was high, and CT findings demonstrated mediastinal, hilar and abdominal lymphadenopathy.  Ref: 12692945 Nihon Kokyuki Gakkai Zasshi, 2002
- Immunosuppression protocol anti IL 2  [ **IL2** ]  receptor  [ **IL2RB** ]  antibodies, steroids, mycophenolate mofetil MMF.  Ref: 12876872 Acta Med Croatica, 2003
- OBJECTIVE The present study was conducted to investigate whether the serum levels of interleukin 6 IL 6, soluble IL 2  [ **IL2** ]  receptor  [ **IL2RB** ]  sIL 2R and sIL 6R are associated with the morphological appearance of rheumatoid arthritis RA.  Ref: 12673891 Clin Exp Rheumatol< MedlineTA>Clin Exp Rheumatol, 2003
- The expression of genes encoding interleukin 2  [ **IL2** ]  IL 2  [ **IL2** ]  and IL 2  [ **IL2** ]  receptor  [ **IL2RB** ]  IL 2R gamma also depends on biotin.  Ref: 12730407 J Nutr, 2003
- Basiliximab interferes with the detection of soluble IL 2  [ **IL2** ]  receptor  [ **IL2RB** ]  by the Immulite Immunoassay system.  Ref: 12667677 J Immunol Methods, 2003
- BACKGROUNDTo define whether immunosuppressive agents that block the interleukin IL 2  [ **IL2** ]  pathway could prevent activation induced cell death of activated T cells in the graft, we measured expression of IL 2  [ **IL2** ] , IL 2  [ **IL2** ]  receptor  [ **IL2RB** ]  alpha chain CD25, IL 15, Fas, and Fas ligand by real time reverse transcription polymerase chain reaction in cardiac allografts.METHODSWe characterized the phenotype of the infiltrating cells CD3, CD68, CD25 by immunohistochemistry.  Ref: 12777860 Transplantation, 2003
- Evaluation of the serum level of IL 6, C reactive protein, soluble IL 6 receptor sIL 6R, and soluble IL 2  [ **IL2** ]  receptor  [ **IL2RB** ]  sIL 2R, together with the activity exerted by IL 3 and IL 4 on some cellular subsets, may constitute an additional element in the differential diagnosis of borderline cases.  Ref: 12733143 Cancer, 2003
- We investigated a possible relation between prolactin receptor PRL R or IL 2  [ **IL2** ]  receptor  [ **IL2RB** ]  alpha IL 2Ralpha, CD25 expression on circulating T lymphocytes and their apoptosis in patients with breast cancer.  Ref: 12698200 Br J Cancer, 2003
- INTERVENTION S Interleukin IL 2  [ **IL2** ]  receptor  [ **IL2RB** ] , IL 6, IL 8, and tumor necrosis factor alpha TNF alpha determination by immunoradiometric assay.  Ref: 12749440 Fertil Steril, 2003
- Anti CD2 mAb also inhibited activation induced expression of the high affinity IL 2  [ **IL2** ]  receptor  [ **IL2RB** ]  IL 2R.  Ref: 12744772 J Interferon Cytokine Res, 2003
- We found a defective T cell response in malnourished children, as indicated by diminished levels of circulating total CD3, helper CD4, IL 2  [ **IL2** ]  receptor  [ **IL2RB** ]  bearing CD4 CD25 and memory helper T cell responses CD4 CD45RO in keeping with the decreased specific IgE levels against Ascaris lumbricoides.  Ref: 12848201 J Trop Pediatr, 2003
- Serum LDH and soluble IL 2  [ **IL2** ]  receptor  [ **IL2RB** ]  were high in titers 552 U l and 90 U l, normal range 145 519.  Ref: 12820543 Rinsho Shinkeigaku< MedlineTA>Rinsho Shinkeigaku, 2003
- Laboratory examination showed elevated levels of serum LDH and soluble IL 2  [ **IL2** ]  receptor  [ **IL2RB** ] , and hypercalcemia.  Ref: 12692982 Rinsho Ketsueki, 2003
- Blockade of intragraft IL 2  [ **IL2** ]  receptor  [ **IL2RB** ]  alpha by basiliximab is insufficient to prevent activation of liver graft infiltrating cells.  Ref: 12727469 Transpl Immunol, 2003
- We report a 70 year old woman with human adjuvant disease who developed lymphoma associated HPS LAHPS and elevated serum soluble interleukin IL 2  [ **IL2** ]  receptor  [ **IL2RB** ] .  Ref: 12739046 Rheumatol Int, 2003
- Addition of IL 12 to this coculture prevented apoptosis of the CD4 T cells, which was associated with up modulation of IL 2  [ **IL2** ]  receptor  [ **IL2RB** ]  beta chain expression.  Ref: 12883484 Hepatology, 2003
- The suppressive activity of CD8 CD25 thymocytes was abrogated by a mixture of anti CTLA 4 and anti TGF beta1 antibodies and it was mediated by their ability to inhibit the expression of the IL 2  [ **IL2** ]  receptor  [ **IL2RB** ]  alpha chain on target T cells.  Ref: 12893750 BloodBlood, 2003
- METHODS AND RESULTS A study of a humanized monoclonal antibody against the high affinity IL 2  [ **IL2** ]  receptor  [ **IL2RB** ]  daclizumab was performed in 70 adult, cardiac transplant recipients.  Ref: 12660501 Transplantation, 2003
- We observed that eosinophils from most patients with hypereosinophilia express the alpha chain of the IL 2  [ **IL2** ]  receptor  [ **IL2RB** ]  CD25,.  Ref: 12672048 Eur J Immunol, 2003
- Parameters of cellular and humoral immunity differential blood count, T cell markers CD2, CD3, CD4, and CD8, B cell markers CD19 and CD20, monocyte markers CD13 and CD14, NK cell marker CD16, activation markers CD25, CD26, CD69 and HLA DR, and cytokines IL 1 receptor antagonist IL 1RA, IL 2  [ **IL2** ] , soluble IL 2  [ **IL2** ]  receptor  [ **IL2RB** ]  sIL 2R, IL 6, IL 10, and TGFbeta were measured in peripheral venous blood.  Ref: 12779014 Folia Biol Praha , 2003
- Most of the 22 interleukin interleukin receptor genes were shown to be highly expressed in preeclamptic placenta, while the expression of IL 2  [ **IL2** ]  receptor  [ **IL2RB** ]  IL 2  [ **IL2** ]  R alpha, GenBank X01057 gene in preeclamptic placenta was comparatively lower than that in normal placenta.  Ref: 12747232 J Perinat Med, 2003
- Although the effects of mitogens on the synthesis of interleukin 2  [ **IL2** ]  IL 2  [ **IL2** ]  and IL 2  [ **IL2** ]  receptor  [ **IL2RB** ]  IL 2r have been described, a detailed in situ analysis of the spatio temporal changes of the expression of the IL 2  [ **IL2** ]  gene and the three IL 2r components in lymphoid tissues is still missing.  Ref: 12890448 Cytokine, 2003
- c the ability of apoE to inhibit the proliferation of T lymphocytes by internalization of the IL 2  [ **IL2** ]  receptor  [ **IL2RB** ] .  Ref: 12769659 Curr Drug Targets Cardiovasc Haematol Disord, 2001
- Most investigated Treg cells are CD4 pos cells expressing the alpha chain of the IL 2  [ **IL2** ]  receptor  [ **IL2RB** ]  CD25.  Ref: 12848995 Autoimmun Rev, 2002
- Plasticity of IL 2  [ **IL2** ]  and IL 2  [ **IL2** ]  receptor  [ **IL2RB** ]  chains in rat lymphoid tissues in situ after stimulation with staphylococcal enterotoxin A.  Ref: 12890448 Cytokine, 2003
- Our study was designed to determine whether a hemodialysis session leads to an acute substantial alteration in the plasma levels of the proinflammatory cytokines interleukin IL 6, IL 1beta and tumor necrosis factor TNF alpha, the T lymphocyte activation factor soluble IL 2  [ **IL2** ]  receptor  [ **IL2RB** ]  sIL 2R, and an inflammation mediator and chemotactic granulocyte factor, IL 8, in end stage renal disease patients receiving chronic intermittent HD.  Ref: 12745544 Mediators Inflamm, 2003
- There was no significant expression of IL 2  [ **IL2** ] , IL 2  [ **IL2** ]  receptor  [ **IL2RB** ] , IL 4, TNFalpha, or IFNgamma.  Ref: 12826912 Pancreas, 2003
- Anti lymphocyte antibodies ATG deplete circulating lymphocytes while selective monoclonal antibodies are directed against IL 2  [ **IL2** ]  receptor  [ **IL2RB** ]  thus reducing the rate of proliferation of activated T cells.  Ref: 12835079 Mol Immunol, 2003
